# Deep Learning for Detecting Subretinal Fluid and Discerning Macular Status by Fundus Images in Central Serous Chorioretinopathy

**DOI:** 10.3389/fbioe.2021.651340

**Published:** 2021-11-05

**Authors:** Fabao Xu, Shaopeng Liu, Yifan Xiang, Zhenzhe Lin, Cong Li, Lijun Zhou, Yajun Gong, Longhui Li, Zhongwen Li, Chong Guo, Chuangxin Huang, Kunbei Lai, Hongkun Zhao, Jiaming Hong, Haotian Lin, Chenjin Jin

**Affiliations:** ^1^ State Key Laboratory of Ophthalmology, Zhongshan Ophthalmic Center, Sun Yat-sen University, Guangzhou, China; ^2^ School of Computer Science, Guangdong Polytechnic Normal University, Guangzhou, China; ^3^ School of Medical Information Engineering, Guangzhou University of Chinese Medicine, Guangzhou, China; ^4^ Center of Precision Medicine, Sun Yat-sen University, Guangzhou, China

**Keywords:** deep learning, central serous chorioretinopathy, subretinal fluid, serous retinal detachment, fundus image

## Abstract

Subretinal fluid (SRF) can lead to irreversible visual loss in patients with central serous chorioretinopathy (CSC) if not absorbed in time. Early detection and intervention of SRF can help improve visual prognosis and reduce irreversible damage to the retina. As fundus image is the most commonly used and easily obtained examination for patients with CSC, the purpose of our research is to investigate whether and to what extent SRF depicted on fundus images can be assessed using deep learning technology. In this study, we developed a cascaded deep learning system based on fundus image for automated SRF detection and macula-on/off serous retinal detachment discerning. The performance of our system is reliable, and its accuracy of SRF detection is higher than that of experienced retinal specialists. In addition, the system can automatically indicate whether the SRF progression involves the macula to provide guidance of urgency for patients. The implementation of our deep learning system could effectively reduce the extent of vision impairment resulting from SRF in patients with CSC by providing timely identification and referral.

## Introduction

As the fourth most common nonsurgical retinopathy, central serous chorioretinopathy (CSC) is an idiopathic ophthalmopathy in which the neurosensory retina is often detached in the central macular region due to serous leakage from defects of the retinal pigment epithelium, causing a condition where fluid accumulates under the retina and thus causes a visual impairment (Wang et al., 2008; Manayath et al., 2018). In Western countries, such as the United States, a population-based study reported that annual age-adjusted incidences of CSC from 1980 to 2002 were 9.9 and 1.7 per 100,000 in men and women, respectively, in a predominantly Caucasian population (van Rijssen et al., 2019). In Eastern countries, however, pachychoroid diseases, such as CSC and polypoidal choroidal vasculopathy, have been considered to be more prevalent than in Caucasian populations (Wong et al., 2016; van Rijssen et al., 2019). Although CSC often causes irreversible visual disability in its later stage, the early diagnosis and timely and proper treatment, such as photodynamic therapy and other laser therapies, can improve the rate of complete absorption of subretinal fluid (SRF) and lead to a satisfactory prognosis of CSC (van Rijssen et al., 2019).

However, identifying all the serous retinal detachments (SRDs) at an early stage remains challenging, as a low volume of SRF often exists asymptomatically and leads to atrophy of the outer layers of the retina (Wong et al., 2016; Xu et al., 2020). Fundus fluorescein angiography (FFA) and optical coherence tomography (OCT) are more sensitive examinations for detecting CSC in clinical work. However, these imaging methods are still not widely available and expensive, especially in some less developed countries and regions (Daruich et al., 2015; Manayath et al., 2018; Zhen et al., 2020). In addition, FFA, as an invasive examination, sometimes leads to severe allergic reactions such as nausea and shock caused by fluorescent dye, which is not suitable for routine detection of SRD (Soomro et al., 2018; He et al., 2020). In the past, there have been some efforts on assessing CSC based on FFA and OCT (Narendra Rao et al., 2019; Zhen et al., 2020); however, considering the clinical practicality, a fundus photograph is the best imaging manner to routinely detect status and severity of patients with CSC. Unfortunately, it is not easy, even for experienced ophthalmologists, to reliably identify CSC on fundus photography. If a computer tool is available to automatically assess the status and severity of patients with CSC using a fundus photograph, an ophthalmologist can perform a timely intervention to avoid the possibility of severe poor prognosis. Consequently, it is essential to develop an intelligent screening approach to detect SRF at an early stage of CSC.

In addition to the presence or absence of SRF, the location of SRF is also a major factor affecting prognosis and treatment (Daruich et al., 2015; Manayath et al., 2018). The presence of macula-on SRF is a potential indicator of the urgency of intervention and the central visual prognosis after treatment, indicating that the macula-on SRF patient needs a more urgent intervention than those with macula-off SRF (Mrejen et al., 2019; Yu et al., 2019). Therefore, to assess the patient’s condition in more detail, we aimed to develop and evaluate a cascaded artificial intelligence (AI) system for detecting SRF and discerning the macular status in patients with CSC based on the fundus photograph.

## Materials and Methods

**FIGURE 1:**
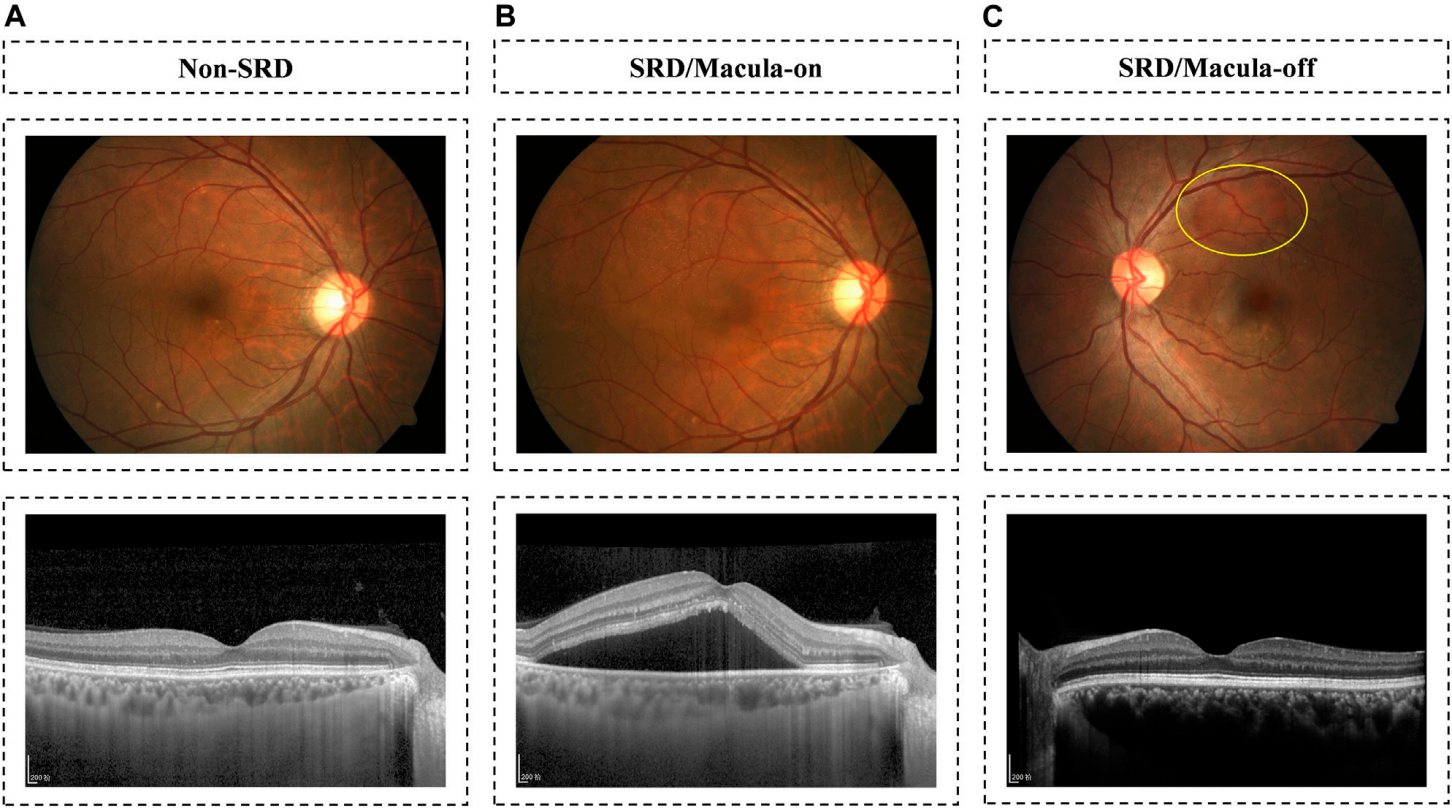
Fundus image examples with OCT-verified CSC. Examples of three CSC states: non-SRD on the panel **(A)**; macula-on SRD on the panel **(B)**; and macula-off SRD in the panel **(C)**.

To develop the cascaded AI system, a total of 12,532 fundus photographs were retrospectively obtained from CSC patients presenting for retinopathy examinations or undergoing a routine ophthalmic health evaluation between February 2015 and January 2020 at the Zhongshan Ophthalmic Center (ZOC) using Zerss (FF-450plus), Topcon (TRC−NW8), and Newvision (RetiCam3100) fundus cameras with 30 or 50° fields of view. For each patient with CSC enrolled, we have both fundus photographs and their corresponding OCT images. We used the OCT images to determine the presence or absence of SRF (Figure 1). An experienced retinal specialist (Chenjin Jin) was responsible for reviewing OCT examinations. All privacy information was removed, and all images were deidentified before transfer to research investigators. Our ethics committee ruled that written informed consent was not required because of the retrospective nature of our study and all the images were fully anonymized. This study was approved by the Institutional Review Board of ZOC, Sun Yat-sen University, and adhered to the tenets of the Declaration of Helsinki.

### Image Classification and the Reference Standard

**FIGURE 2:**
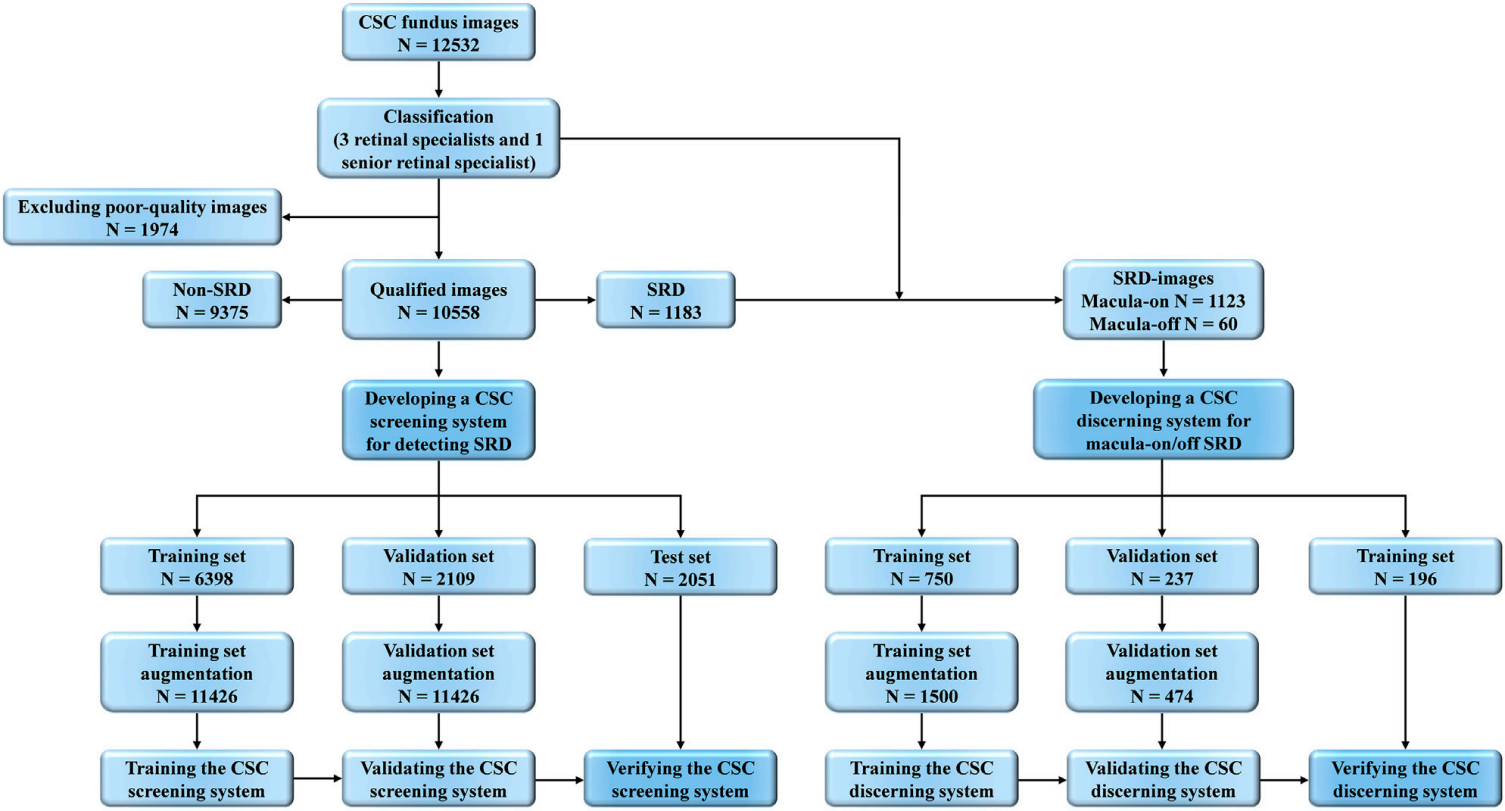
Overall Study Workflow. Workflow of developing deep learning systems for identifying SRD and discerning macula-on/off SRD based on fundus images. SRD, serous retinal detachment; CSC, central serous chorioretinopathy.

According to OCT images, all fundus photographs were classified into SRD and non-SRD. Then, we further classified SRD images into macula-on SRD and macula-off SRD according to whether SRF extended to involve the fovea within 300 microns. Image quality was defined as follows: 1) high quality referred to images without any problems; 2) relatively high quality referred to images with slight deficiencies in focus, illumination, or topo-artifacts, but the region of optic disc and macula could still be identified completely; 3) medium quality referred to images with an obscured view of the image (smaller than one-third of the image), but part of the SRD region could still be identified, and the region of the optic disc and the macula could be identified; 4) poor quality referred to images that were insufficient for any interpretation (an obscured area over one-third of the image), or the region of the optic disc and the macula could not be identified. To ensure the accuracy of image classification, all anonymous fundus photographs were classified separately by three board-certified retinal specialists with at least 5 years of clinical experience (Zhongwen Li, Fabao Xu, and Longhui Li). Any disagreement was arbitrated by another senior retinal specialist with more than 30 years of clinical experience (Chenjin Jin). Figure 2 illustrates the workflow of the study.

### Data Preprocessing and Augmentation

The image data were standardized and normalized before deep learning model training. Firstly, all the images were resized to 512 × 512 pixels, where each pixel value was further rescaled to the range of 0–1. Secondly, to build deep learning models adapting different kinds of variations in fundus images, three data augmentation operations were deployed to expand our image datasets artificially, including random rotation (at the angle of 90, 180, and 270°, respectively), cropping (512 × 512 to 320 × 320, and then resize back to 512 × 512), and flipping.

### Deep Learning System Development

**FIGURE 3:**
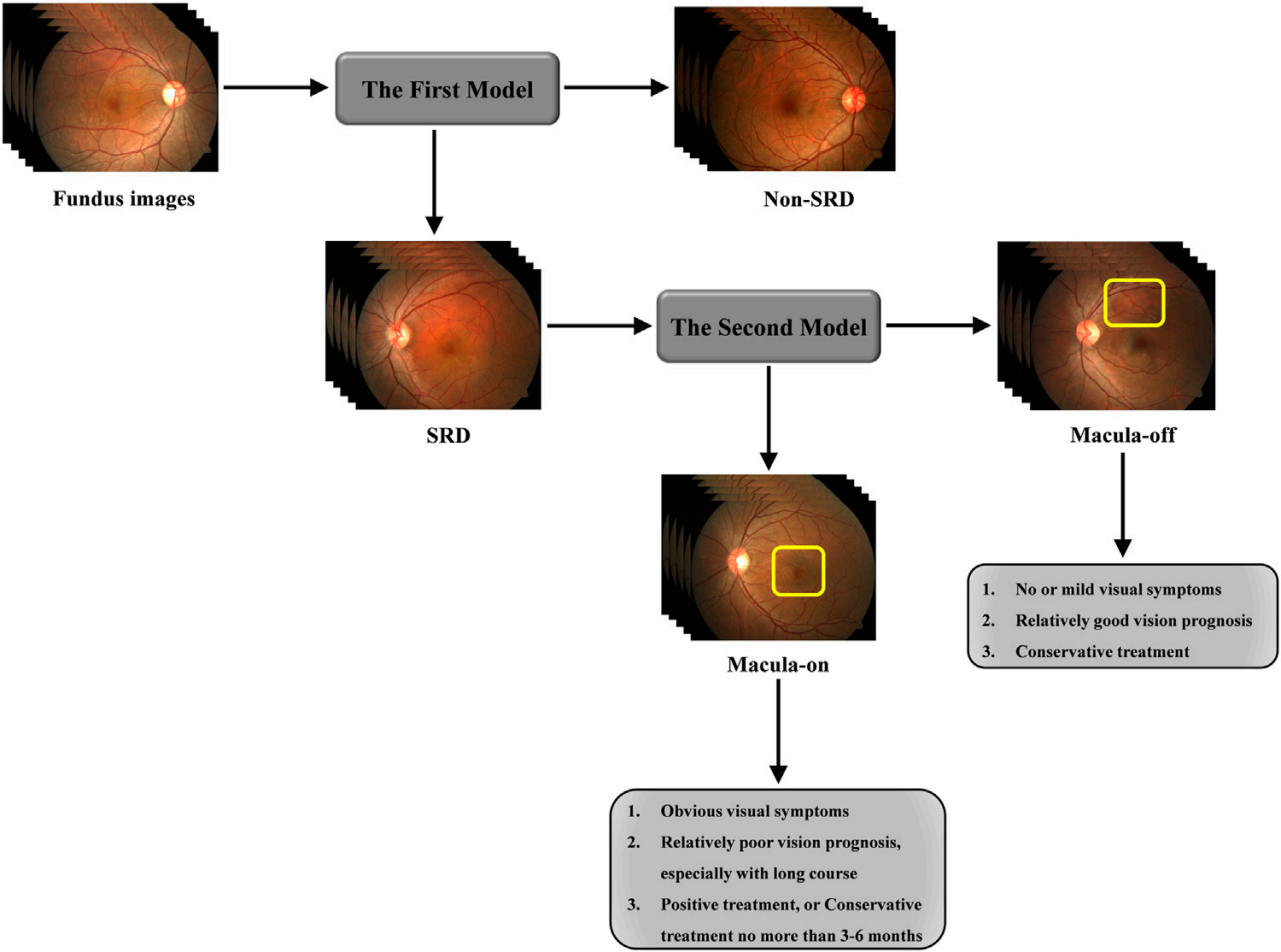
Framework of cascaded deep learning system and its corresponding clinical application. CSC often has a halo-like appearance in macula regions due to SRD. Top left, A fundus image of a normal subject without SRD; Top middle, A fundus image of a patient with SRD at macula; Top right, A fundus image of a patient with SRD away from macula (circled in yellow). SRD, serous retinal detachment; CSC, central serous chorioretinopathy.

To effectively detect SRD and discern macular status by fundus images, a cascaded architecture of convolutional neural networks (CNNs) was deployed. To be specific, cascaded systems have two separate CNN models. The model in the first stage focuses on the early identification of SRD by fundus images, whereas the model in the second stage focuses on further classifying SRD images into macula-on SRD or macula-off SRD (shown in Figure 3).

Two models were trained separately using a state-of-the-art CNN model, EfficientNet-B0 (Zhao et al., 2020). EfficientNet is a powerful CNN model proposed by Google in recent years, which can automatically scale network height, width, and resolution to achieve an efficient classification effect. To train the first model, all the fundus images collected were used with labels of SRD or non-SRD. As for the second model, the SRD images were used with labels of macula-on SRD or macula-off SRD. To make models more reliable, a whole data set was randomly divided into three non-overlapping subsets, namely, training set, validation set, and testing set. The training set was used to produce candidate models; the validation set was used to assess these models and help determine the optimal ones as the final applied models. The final models were then evaluated using the testing set. We performed image standardization before deep feature learning, and data augmentation was applied to increase the diversity of the dataset and thus reduce the chance of overfitting. The implementation and training details of the models are as follows. Firstly, transfer learning from the pretrained EfficientNet-B0 was adopted to improve the training effect. Secondly, we use the focal loss as loss functions of the two EffecientNet models considering that the sample sizes for different classes are unbalanced. As is universally acknowledged, the focal loss function is specially designed for training problems with class imbalance. Therefore, the models can pay more attention to the samples in the minority class in the training process. To be concrete, we set the alpha parameter in the focal loss function to be 0.5 in both models. Lastly, we use Adam as the optimizer in training EfficientNet and set the learning rate to be 0.003, the learning rate decay factor as 0.99, the total number of epochs to be 50, and the batch size to be 16.

### Model Evaluation

We evaluated the performance of the developed AI system using the independent testing data set, among which no image was involved in the training set. We accessed nonparametric receiver operating characteristic analysis on the testing data set and calculated the 95% confidence intervals. The sensitivity, specificity, and accuracy of the systems for detecting SRD and discerning the macular status were also computed. We asked three retinal specialists (Cong Li, Hongkun Zhao, and Lijun Zhou) who had 3, 5, and 10 years of experience, respectively, to independently assess CSC status in the testing data. Then, we compared their performance with the deep learning models.

## Results

### Demographic Characteristics

As shown in Figure 1, 12,532 fundus photographs from 568 CSC patients (mean age 45.23 ± 7.45 years; range, 31–72 years) and 4,126 subjects (mean age 46.41 ± 8.84 years; range, 12–78 years) presenting for retinopathy examinations or undergoing a routine ophthalmic health evaluation were labeled for SRD or non-SRD. In the quality control phase, 1,974 poor-quality images of 51 CSC patients and 1,028 normal subjects were deleted due to the opacity of the refractive media or artifacts (e.g., images without decipherable optic disc or fovea, arc defects over one-third of the area, dust spots on the optic disc and/or fovea, or images with incorrect focus). The first AI system designed to identify SRD was developed using 10,558 fundus photographs, 1,183 of which were classified as SRD, whereas the remaining 9,375 images were classified as non-SRD. All eligible images were randomly divided into three sets in a patient-level (no overlapping patients), with 60% (6,398 images) as a training set, 20% (2,109 images) as a validation set, and 20% (2051 images) as a test set. Then, the second AI system was developed using 1,183 SRD images to discern macula-off SRD from macula-on SRD, with 60% (750 images) as a training set, 20% (237 images) as a validation set, and 20% (196 images) as a test set. The functions of the two AI systems are shown in Figure 3. The numbers of labels and demographic data in the training, validation, and test sets are shown in Table 1.

**TABLE 1:** Patient demographics.

Characteristic	CSC group	Normal group
Patients (*n*)	568	4,126
Images (SRF label)	4,316 (1,183)	6,242
Age (years)	45.23 ± 7.45	46.41 ± 8.84
Males (%)	482 (84.86)	2,105 (51.02)
Train set (SRF label)	2,566 (685)	3,823
Validation set (SRF label)	906 (271)	1,203
Test set (SRF label)	844 (227)	1,207

CSC, central serous chorioretinopathy.

### Performance of Deep Learning Models

**FIGURE 4:**
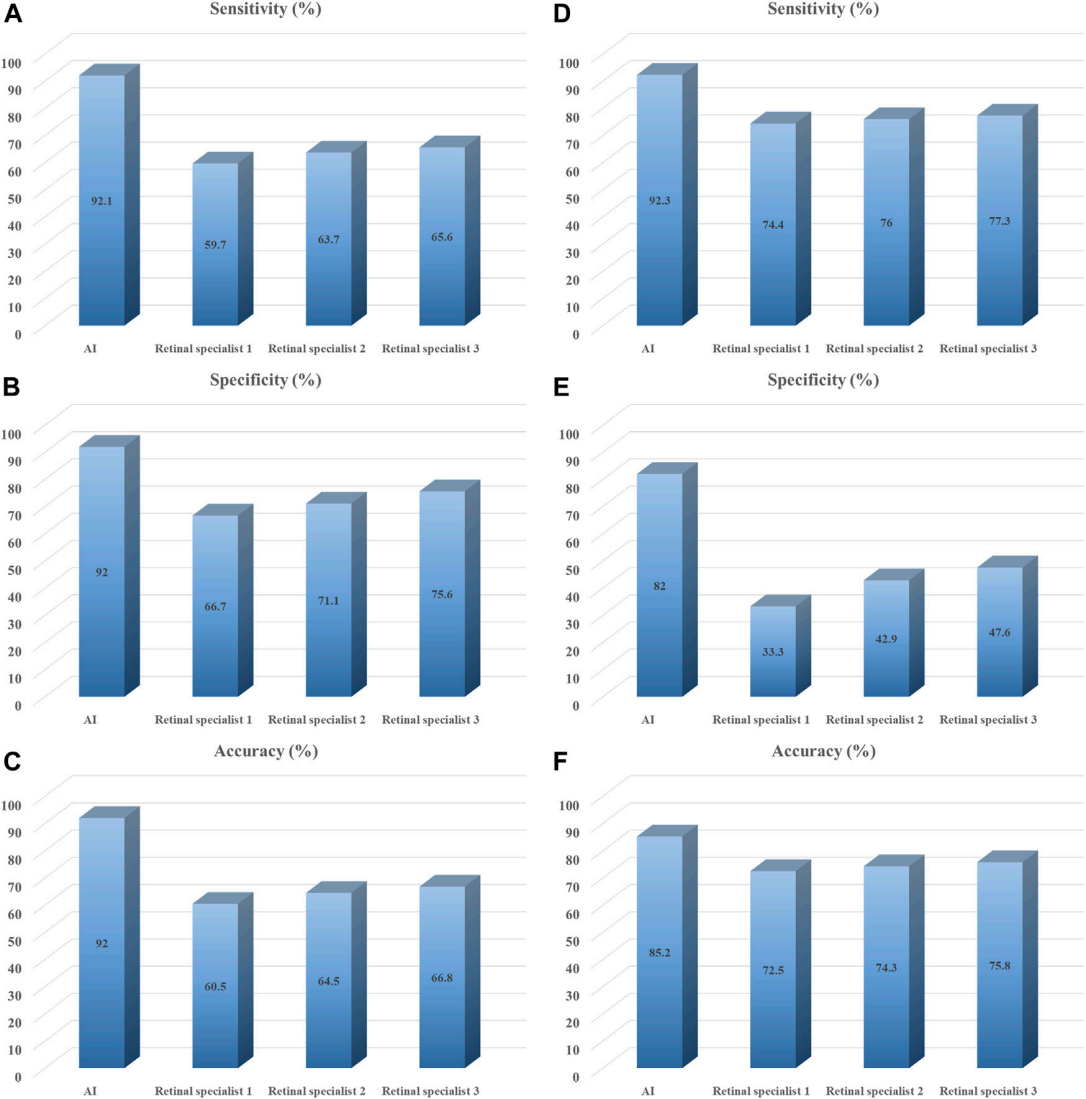
Performance of deep learning models *vs.* retinal specialists in test set. Summary of classification results by deep learning models and three retinal specialists on testing data set. **(A–C)** Identification performance of SRD; **(D–F)** Discern performance of macula-on SRD. Retinal specialist 1 with 3 years of working experience at a physical examination center; retinal specialist 2 with 5 years of working experience at a physical examination center; retinal specialist 3 with 10 years of working experience at a physical examination center.

**FIGURE 5:**
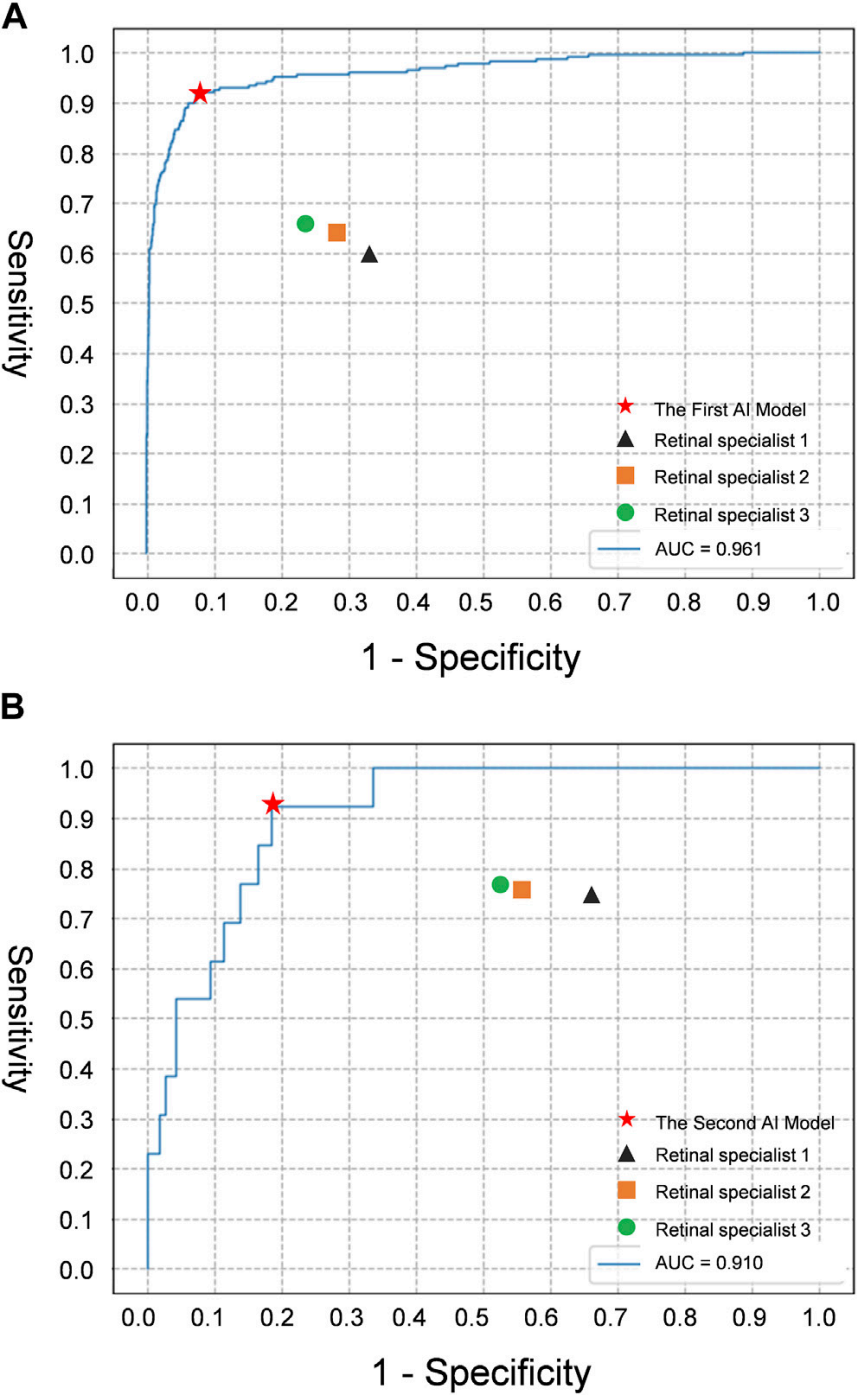
The receiver operating characteristic curve of the deep learning models for evaluating CSC depicted on fundus images, compared with retinal specialists’ performance. **(A)** Detection performance of SRD; **(B)** Detection performance of macula-on SRD. AUC area under the receiver operating characteristic curve; Retinal specialist 1, 3 years of working experience; retinal specialist 2, 5 years of working experience; retinal specialist 3, 10 years of working experience.

The performance of the AI models and general ophthalmologists to detect SRD and discern the macular status is shown in Table 2. For SRD detection, the retinal specialist 1 with 3 years of experience had a sensitivity of 59.7% and a specificity of 66.7%, the retinal specialist 2 with 5 years of experience had a sensitivity of 63.7% and a specificity of 71.1%, and the retinal specialist 3 with 10 years of experience had a sensitivity of 65.6% and a specificity of 75.6%, whereas the first AI model had a sensitivity of 92.1% and a specificity of 92.0% (Figures 4A–C) with an area under the curve (AUC) of 0.961 (Figure 5A).

**TABLE 2:** Performance of the AI systems *vs.* general ophthalmologists in the test sets.

SRD/non-SRD	Sensitivity (95% CI)	Specificity (95% CI)	Accuracy (95% CI)
AI system	0.921 (0.846, 0.996)	0.920 (0.845, 0.995)	0.920 (0.845, 0.995)
Retinal specialist 1	0.597 (0.549, 0.645)	0.667 (0.621, 0.713)	0.605 (0.557, 0.653)
Retinal specialist 2	0.637 (0.590 0.684)	0.711 (0.667, 0.755)	0.645 (0.598, 0.692)
Retinal specialist 3	0.656 (0.609, 0.703)	0.756 (0.714, 0.798)	0.668 (0.622, 0.714)
**Macula on/off**			
AI system	0.923 (0.849, 0.997)	0.820 (0.698, 0.916)	0.852 (0.708, 0.930)
Retinal specialist 1	0.744 (0.701, 0.787)	0.333 (0.287, 0.379)	0.725 (0.681, 0.769)
Retinal specialist 2	0.760 (0.718, 0.802)	0.429 (0.380, 0.478)	0.743 (0.700, 0.786)
Retinal specialist 3	0.773 (0.732, 0.814)	0.476 (0.427, 0.525)	0.758 (0.716, 0.800)

AI, artificial intelligence. Ophthalmologist 1 with 3 years of working experience at a physical examination center; ophthalmologist 2 with 5 years of working experience at a physical examination center; ophthalmologist 3 with 10 years of working experience at a physical examination center. CI, confidence interval.

For discerning macula-on SRD from macula-off SRD, the retinal specialist 1 with 3 years of experience had a sensitivity of 74.4% and a specificity of 33.3%, the retinal specialist 2 with 5 years of experience had a sensitivity of 76.0% and a specificity of 42.9%, and the retinal specialist 3 with 10 years of experience had a sensitivity of 77.3% and a specificity of 47.6%, whereas the second AI model had a sensitivity of 92.3% and a specificity of 82.0% (Figures 4D–F) with an AUC of 0.910 (Figure 5B).

## Discussion

In this study, we developed a cascaded deep learning system containing two models based on 11,087 fundus images and verified its feasibility. The first deep learning model was used to identify SRD and showed robust performance (AUC 0.961, a sensitivity of 92.1%, and a specificity of 92.0%). The second model used to discern macula-on SRD from macula-off SRD also exhibited ideal performance (AUC 0.910, a sensitivity of 92.3%, and a specificity of 82.0%). The deep learning models performed better than the retinal specialists in both tasks. These results validate that our deep learning models provide an objective SRD detection with high accuracy and efficiency in patients with CSC, while also precisely determining whether the macula is involved. Overall, the intelligent system achieved a better performance in classification, demonstrating the potential of the deep learning technology in evaluating CSC based on fundus photography.

As shown, the specificities of both deep learning models were higher than those of the retinal specialists (Table 2). As high sensitivity and specificity are a prerequisite for a screening tool and can reduce the workload and medical costs by avoiding the need for further examinations of evidently normal eyes. High-dose hormone shock therapy and long-term maintenance dose corticosteroids intake often lead to secondary CSC (Tsai et al., 2014; Daruich et al., 2015; Manayath et al., 2018), so this system can be used to screen CSC as a part of the ophthalmic routine evaluations in common corticosteroids therapy departments, such as endocrinology, gastroenterology, and rheumatology, that were lacking ophthalmologists or be deployed in hospitals with a large number of patients to assist ophthalmologists. Advances in OCT have enabled observation of the SRD, but not all hospitals and clinics possess OCT devices, especially in developing countries. If SRDs of patients with CSC could be detected by deep learning models with a fundus image instead of an OCT device, we could detect the status of patients more conveniently at a lower cost. Besides, the models could be used to alert patients to the emergency of SRD.

There have been a number of deep learning architectures developed for fundus disease evaluation, and they can be largely grouped into three different categories based on their objectives, including screening (Gulshan et al., 2016; Voets et al., 2019), prognosis prediction (Poplin et al., 2018), and computer-assisted system for diagnosis and treatment (Khojasteh et al., 2018; Shankaranarayana et al., 2019). AI is playing an increasingly important role in medical activities, such as diabetes screening systems, which has begun to serve ophthalmic health evaluations in physical examination centers or community hospitals lacking ophthalmologists, or be deployed in hospitals with a large number of patients for ophthalmologists’ assistance (Ting et al., 2017; Ting et al., 2019). Previously, Narendra Rao et al. (2019) reported a deep learning-based system for automatic detection and segmentation of sub-retinal fluid in CSC by OCT images. However, limited work has been dedicated to identifying CSC using color fundus. Compared with their study, the system developed in our study can detect SRDs based on fundus images and evaluate the emergency of CSC, which is more conformed to clinical application. The availability of such a tool could be helpful to ophthalmologists as a second eye to timely and accurately detect CSC with the wide-applied and noninvasive color fundus photography. In particular, it may reduce unnecessary fluorescein angiography and/or OCT examinations, which may involve adverse effects or additional cost and often not be available in developing areas.

Several limitations exist in this study. First, all follow-up data for a patient at different times were included, the variety and the number of the images were limited, and all these images were acquired from a single institution. We expect that the performance may significantly improve when using a large, diverse data set for training the deep learning network and dedicating additional effort to optimize the training parameters. Second, we did not verify whether, and to what extent, different fundus camera equipment may affect CSC assessment. It is well known that the standardization of train data remains a key point to the development of AI, such as image scope, light exposure, focus, and sharpness. Finally, the training set and test set did not distinguish between acute and chronic CSCs due to a lack of reference standards (Manayath et al., 2018). Despite the importance of defining a recognized classification system for CSC, no consensus has been reached so far. CSC is commonly divided into two categories based on the duration of symptoms (6 months), acute and chronic CSCs (Manayath et al., 2018). However, many of the enrolled patients were followed for more than 6 months, but their imaging findings may not change obviously before or after this demarcation line. Therefore, we did not conduct a subgroup analysis of acute and chronic CSCs in this study.

In conclusion, the present study verifies that our robust cascaded deep learning system can be applied to detect SRF and discern macula status efficiently in patients with CSC. Furthermore, our deep learning system provides a template to patients with fundus diseases, which are characterized by SRF progression. Due to the convenience and versatility of fundus images, a future effort is desirable to make it available to the clinical practice for initial CSC screening in common therapy departments and CSC follow-up departments. Prospective clinical studies to evaluate the cost-effectiveness and the performance of this system in real-world settings are needed.

## Data Availability

The data analyzed in this study is subject to the following licenses/restrictions: The data are temporarily not available for privacy reasons but can be obtained for legitimate reasons in consultation with the corresponding author. Requests to access these datasets should be directed to HL, haot.lin@hotmail.com.
